# Koilocytes due to HPV in the urine sediment

**DOI:** 10.1590/2175-8239-JBN-2019-0158

**Published:** 2020-04-27

**Authors:** José Antonio Tesser Poloni, Gisele Meinerz, Cássia Ferreira Braz Caurio, Alessandro Comarú Pasqualotto

**Affiliations:** 1 Universidade do Vale do Rio dos Sinos, Faculdades de Farmácia e Biomedicina, São Leopoldo, RS, Brasil.; 2 Controllab, Rio de Janeiro, RJ, Brasil.; 3 Santa Casa de Misericórdia de Porto Alegre, Porto Alegre, RS, Brasil.; 4 Santa Casa de Misericórdia de Porto Alegre, Laboratório de Análises Clínicas Carlos Franco Voegeli, Porto Alegre, RS, Brasil.; 5 Universidade Federal de Ciências da Saúde de Porto Alegre, Faculdade de Medicina, Departamento de Doenças Infecciosas, Porto Alegre, RS, Brasil.; 6 Santa Casa de Misericórdia de Porto Alegre, Laboratório de Biologia Molecular, Porto Alegre, RS, Brasil.

**Keywords:** Papillomaviridae, Urinalysis, Papillomaviridae, Urinálise

## Abstract

Koilocytes are the hallmark of human papillomavirus (HPV) infection and can be observed during routine cytology tests stained by Papanicolaou. However, the test is not part of the routine urinalysis report. Here we describe a case on HPV subtype 6 infection diagnosed after finding koilocytes in fresh and unstained urine sediment of a kidney allograft recipient male patient.

## RESUMO

Os coilócitos são um sinal característico da infecção por papilomavírus humano (HPV) e podem ser observados durante testes citológicos de rotina, corados pelo exame de Papanicolau. Contudo, o exame não faz parte do laudo de rotina da urinálise. O presente artigo descreve um caso de infecção por HPV subtipo 6, diagnosticada após a identificação de coilócitos em sedimento urinário fresco não corado de um paciente do sexo masculino receptor de aloenxerto renal.

## CASE

**Figure 1:**
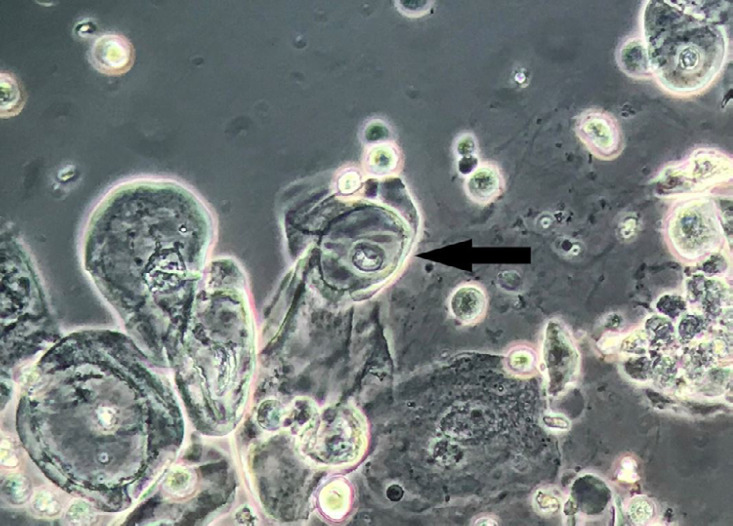
Perinuclear halo in squamous epithelial cell - koilocyte (arrow). Fresh and unstained urine sediment. Phase contrast microscopy. Original magnification 400x.

A 62-year-old man was submitted to kidney transplantation due to polycystic kidney disease in 2017. During a routine follow-up visit, the patient had laboratorial tests performed, including urinalysis. Dipstick test on urine revealed a pH of 5.0, urine-specific gravity of 1.012, 1+ of albumin and 1+ of haemoglobin. Urine microscopy revealed 31-40 squamous epithelial cells per high power field (HPF), >50 white blood cells/HPF, 1-2 red blood cells/HPF and <1 granular casts per low power field. Noteworthy, some squamous epithelial cells presented a clear perinuclear halo (Figure 1; koilocytes) - as commonly observed during routine cervical cytology analysis in Papanicolaou-stained samples in the context of human papillomavirus (HPV) infection^1^^,^ 2. These findings are not usual to occur in fresh and unstained urine sediment, especially in a male patient. After the urinary finding, cystoscopy was performed and revealed lesions in the urethra, wich were cauterized. In an attempt to identify the pathogenic agent, an in-house polymerase chain reaction (PCR) test was performed and confirmed the presence of HPV DNA subtype 6 in the urine sample. HPV6 is a low risk type of HPV, which has rarely been associated to the development of tumours. However, in the context of immunosuppression, HPV6 can cause potentially severe infections. As shown in this case, urine sediment analysis can play a role in the diagnosis of HPV infections.

## References

[1] Altamirano E, Drut R (2008). Koilocytes in urinary cytology in a patient with kidney transplant. Diagn Cytopathol.

[2] Goyal A, Ray N, Chute DJ, Abdul-Karim FW (2014). Significance of cytologic detection of low-grade squamous intraepithelial lesions in urine. J Am Soc Cytopathol.

